# Risk of intracranial haemorrhage and ischaemic stroke after convexity subarachnoid haemorrhage in cerebral amyloid angiopathy: international individual patient data pooled analysis

**DOI:** 10.1007/s00415-021-10706-3

**Published:** 2021-07-17

**Authors:** Isabel Charlotte Hostettler, Duncan Wilson, Catherine Arnold Fiebelkorn, Diane Aum, Sebastián Francisco Ameriso, Federico Eberbach, Markus Beitzke, Timothy Kleinig, Thanh Phan, Sarah Marchina, Romain Schneckenburger, Maria Carmona-Iragui, Andreas Charidimou, Isabelle Mourand, Sara Parreira, Gareth Ambler, Hans Rolf Jäger, Shaloo Singhal, John Ly, Henry Ma, Emmanuel Touzé, Ruth Geraldes, Ana Catarina Fonseca, Teresa Melo, Pierre Labauge, Pierre-Henry Lefèvre, Anand Viswanathan, Steven Mark Greenberg, Juan Fortea, Marion Apoil, Marion Boulanger, Fausto Viader, Sandeep Kumar, Velandai Srikanth, Ashan Khurram, Franz Fazekas, Veronica Bruno, Gregory Joseph Zipfel, Daniel Refai, Alejandro Rabinstein, Jonathan Graff-Radford, David John Werring

**Affiliations:** 1 Stroke Research Centre, University College London, National Hospital of Neurology and Neurosurgery, Institute of Neurology, Queen Square, London, WC1N UK; 2 Department of Neurology, Mayo Clinic, Rochester, MN USA; 3 Department of Neurological Surgery, Washington University School of Medicine, St. Louis, MO USA; 4 Institute for Neurological Research, Fleni, Buenos Aires Argentina; 5 Department of Neurology, Medical University of Graz, Graz, Austria; 6 Department of Neurology, Royal Adelaide Hospital, Adelaide, Australia; 7 Department of Neurology, Monash Health and Stroke and Ageing Research Group, Melbourne, Australia; 8 Department of Neurology, Stroke Division, Beth Israel Deaconess Medical Center, Harvard Medical School, Boston, MA USA; 9 Department of Neurology, CHU Caen Normandie, Caen, France; 10 Memory Unit, Department of Neurology, Hospital de la Santa Creu I Sant Pau, Institut Investigació Biomèdica Sant Pau, Universitat Autònoma de Barcelona, Barcelona, Spain; 11 J. Philip Kistler Stroke Research Center, Department of Neurology, Massachusetts General Hospital and Harvard Medical School, Boston, MA USA; 12 Department of Neurology, CHU de Montpellier, Hôpital Gui-de-Chauliac, Montpellier, France; 13 Stroke Unit, Department of Neuroscience, Hospital de Santa Maria, University of Lisbon, Lisbon, Portugal; 14 Department of Statistical Science, UCL, London, WC1E 6BT UK; 15 Neuroradiological Academic Unit, Department of Brain Repair & Rehabilitation, University College London, Institute of Neurology, London, UK; 16 Normandy University, UNICAEN, INSERM U1237, Caen, France; 17 Nuffield Department of Clinical Neurosciences, Oxford University Hospitals, Oxford, UK; 18 Neurology department, Frimley Health Foundation Trust, Camberley, UK; 19 Department of Neuroradiology, CHU de Montpellier, Hôpital Gui-de-Chauliac, Montpellier, France; 20 Department of Neurosurgery, Emory University, Atlanta, GA USA; 21 Department of Medicine, School of Clinical Sciences, Monash University, Melbourne, Australia

**Keywords:** Non-traumatic convexity/convexal/cortical subarachnoid haemorrhage, Intracerebral haemorrhage, Ischemic stroke, Cerebral amyloid angiopathy, Stroke

## Abstract

**Objective:**

To investigate the frequency, time-course and predictors of intracerebral haemorrhage (ICH), recurrent convexity subarachnoid haemorrhage (cSAH), and ischemic stroke after cSAH associated with cerebral amyloid angiopathy (CAA).

**Methods:**

We performed a systematic review and international individual patient-data pooled analysis in patients with cSAH associated with probable or possible CAA diagnosed on baseline MRI using the modified Boston criteria. We used Cox proportional hazards models with a frailty term to account for between-cohort differences.

**Results:**

We included 190 patients (mean age 74.5 years; 45.3% female) from 13 centers with 385 patient-years of follow-up (median 1.4 years). The risks of each outcome (per patient-year) were: ICH 13.2% (95% CI 9.9–17.4); recurrent cSAH 11.1% (95% CI 7.9–15.2); combined ICH, cSAH, or both 21.4% (95% CI 16.7–26.9), ischemic stroke 5.1% (95% CI 3.1–8) and death 8.3% (95% CI 5.6–11.8). In multivariable models, there is evidence that patients with probable CAA (compared to possible CAA) had a higher risk of ICH (HR 8.45, 95% CI 1.13–75.5, *p *= 0.02) and cSAH (HR 3.66, 95% CI 0.84–15.9, *p *= 0.08) but not ischemic stroke (HR 0.56, 95% CI 0.17–1.82, *p *= 0.33) or mortality (HR 0.54, 95% CI 0.16–1.78, *p *= 0.31).

**Conclusions:**

Patients with cSAH associated with probable or possible CAA have high risk of future ICH and recurrent cSAH. Convexity SAH associated with probable (vs possible) CAA is associated with increased risk of ICH, and cSAH but not ischemic stroke. Our data provide precise risk estimates for key vascular events after cSAH associated with CAA which can inform management decisions.

**Supplementary Information:**

The online version contains supplementary material available at 10.1007/s00415-021-10706-3.

## Introduction

Convexity subarachnoid haemorrhage (cSAH) describes non-traumatic subarachnoid bleeding limited to the subarachnoid space over the convexities of the brain that does not extend into the parenchyma, sylvian fissures, ventricles, or basal cisterns [1, 2]. cSAH may present with transient focal neurological episodes (TFNE) of unilateral spreading sensory or motor symptoms [3–5]. In older individuals (over about 60 years), cSAH is often associated with imaging markers of cerebral amyloid angiopathy (CAA) including cortical superficial siderosis (cSS), cerebral microbleeds (CMBs) or both [6]. CAA is characterized by amyloid deposition within pial and cortical penetrating arterioles [7, 8] and is an important cause of symptomatic intracerebral haemorrhage (ICH) [9–11]. An aggregate data meta-analysis from small cohort studies of patients with cSAH and suspected CAA found a high rate of subsequent symptomatic ICH [12] but did not investigate recurrent cSAH, ischemic stroke, or mortality. An understanding of the prognosis for intracranial bleeding and ischemia is important to inform management decisions, including starting or restarting antithrombotic drugs. Moreover, previous studies have not established the time course of these key adverse vascular outcomes.

We therefore performed an international collaborative individual patient data pooled analysis of cohort studies of patients with cSAH and known probable and possible CAA status to increase statistical power and improve the precision of event rate estimates for ICH, symptomatic ischemic stroke, recurrent cSAH and death. We investigated whether neuroimaging markers indicating probable CAA influences the risks of these events.

## Material and methods

### Request for individual patient-data

A repeated systematic literature review (conducted by 2 authors ICH and DW) using PubMed, EMBASE and reference searches identified 21 potentially eligible publications for this individual patient-data pooled analysis, including our own cohort (see Figure e-1) [12]. The search was not restricted by language. As previously described the key words used were “convex* adj4 subarachnoid OR cortical adj4 subarachnoid OR sulc* adj4 subarachnoid”. Of these, 14 groups agreed to contribute individual patient-data of their already published data; if groups included overlapping samples, we included the latest and larger sample making sure no patient was included twice; we thus included data from 13 cohorts [3, 4, 12–22]. A pre-specified protocol and analysis plan was agreed upon among all collaborating centers to ensure uniform definitions for diagnosis and outcome parameters. Clinical and outcome data were collected prospectively and retrospectively at each center as part of ongoing clinical registries. The main inclusion criteria were: non-traumatic symptomatic cSAH (haemorrhage judged acute on CT, MRI or both, limited to the subarachnoid space over the convexities of the brain, not extending into the parenchyma, sylvian fissure, ventricles, or basal cisterns [1]); cSAH attributed to probable or possible CAA (after local investigation and excluding other causes) according to the modified Boston criteria defined by MRI [23]; and available follow-up data. We excluded patients with any underlying alternative cause of cSAH including ruptured aneurysm, arterio-venous-malformation, tumors, reperfusion injuries or hemorrhagic transformation of ischemic stroke, vasculitis, other inflammatory diseases, or reversible cerebral vasoconstriction syndrome (RCVS, the most common cause of cSAH in younger people, defined as cSAH in patients with reversible abnormalities in vessel caliber found on CTA, MRA or DSA [2, 24].

Anonymized data was transferred from participating cohorts to the Stroke Research Centre, UCL Queen Square Institute of Neurology, using a pre-specified data collection sheet. Authors contributed individual patient-data, including patient characteristics, past medical history, medication history and, where available, baseline brain imaging markers and follow-up data. Data was checked for internal consistency with respect̄ to range, and consistency with published reports. Inconsistencies or missing data were reviewed, and attempts were made to resolve any inconsistencies by consensus. In case where missing values are present this is indicated in the tables.

### Definition of outcomes

Symptomatic ICH was defined as acute or subacute onset of neurological symptoms (i.e., occurring within a few days before presentation) with radiological evidence of recent intracerebral haemorrhage (acute blood, perihematomal edema). Recurrent cSAH was defined as acute haemorrhage limited to the subarachnoid space over the convexities of the brain, not extending into the parenchyma, sylvian fissures, ventricles, or basal cisterns [1]. Symptomatic ischemic stroke was defined as acute or subacute focal neurological symptoms attributed to cerebral infarction confirmed by brain imaging. Outcome events were ascertained locally by contributing teams. All events were truncated at 5 years.

### Risk of bias and study quality

We reported our study according to the STROBE guidelines and did the pooled analysis according to the PRISMA guidelines [25, 26]. We assessed all studies for risk of bias and quality using the Cochrane Collaboration tool, which demonstrated a low risk of bias (see Table e-1).

### Radiological data

Probable CAA (versus possible CAA) was diagnosed according to the modified Boston criteria by trained observers at each center. Convexity SAH was not included as a component of the modified Boston criteria [12, 23]. White matter hyperintensities (leukoaraiosis) were measured using the van Swieten scale and divided into “severe” (any score of 2) or non-severe (no score of 2) [27]. Cerebral microbleeds were rated on blood-sensitive sequences (T2* weighted or susceptibility weighted images, SWI) [28]. Cortical superficial siderosis (cSS) was rated on T2*-GRE and SWI sequences [29, 30].

### Statistical analysis

#### Univariable analysis

We estimated the rates of each outcome event using Kaplan Meier survival curves. We investigated the association between the risk of cSAH and each outcome separately using the Cox proportional hazards model with a frailty term to account for differences between study cohorts. The frailty term allows the (absolute) risk to be different in the different studies, i.e., to adjust for unmeasured study-level covariates. We checked the proportional hazards assumption for all analyses by visual inspection of the log–log plot of survival (log cumulative hazard versus log time). If the lines were not parallel, we tested the proportional hazard assumption using Schoenfeld residuals.

#### Multivariable analysis

We performed a pooled multivariable regression analysis for each outcome using a frailty term to account for differences between study cohorts. The multivariable model included the prespecified variables probable CAA and age, as well as variables that had a p value below 0.2 in the univariable analysis for each outcome. As a sensitivity analysis we conducted a competing risk analysis for all of the outcome events. Due to a strong degree of overlap (collinearity), we did not include probable CAA and cortical superficial siderosis in the same model.

Statistical analysis was performed using STATA 15 (StataCorp. 2017. *Stata Statistical Software: Release 15*. College Station, TX: StataCorp LLC).

## Results

We identified 190 eligible patients with cSAH in 13 cohorts published or recruited between 2001 and 2018 with 385 patient-years of follow-up (median follow-up time 1.4 years). The mean age was 74.5 (SD 8.8) and 86 (45.3%) were female. See Table 1 for the overall baseline characteristics and supplementary Table e-2 for individual cohort data. 153 patients (80.5%) fulfilled the modified Boston criteria for probable CAA and 37 (19.5%) for possible CAA. The characteristics of patients by CAA status are shown in supplementary Table e-3.

**Table 1:** Baseline characteristics of the full cohort

	All *N* = 190
Age, mean (SD)	74.5 (8.7)
Female sex	86 (45.3)
Current smoker, *N* (%)	17/169^a^ (10.1)
Current drinker, *N* (%)	22/149 (14.8)
PMH	
HTN, *N* (%)	117 (61.6)
Hypercholesterolemia, *N* (%)	85 (44.7)
DM, *N *(%)	26 (13.7)
AF, *N *(%)	18/186 (9.9)
OAC use, *N *(%)	15/185 (8.1)
Antiplatelet use, *N *(%)	63/185 (34.1)
Statin use, *N *(%)	66/185 (35.7)
Anti HTN use, *N *(%)	104/185 (56.2)
Previous ICH, *N *(%)	24 (12.6)
Previous IS, *N *(%)	21 (11.1)
Symptoms	188/190
Negative, *N *(%)	90 (47.9)
Positive, *N *(%)	58 (30.9)
Both, *N *(%)	27 (14.4)
Headache, *N *(%)	11 (5.9)
Other symptoms, *N *(%)	2 (1.1)
Spreading symptoms, *N *(%)	57/189 (30.2)
Future events	
ICH, *N *(%)	51 (26.8)
Ischemic stroke, *N *(%)	19 (10)
Recurrent cSAH, *N *(%)	39 (20.5)
Restarted on Antiplatelets	54/171 (31.6)
Restarted on OAC	13/169 (7.7)
Death, *N *(%)	31 (16.3)
Probable CAA	153 (80.5)
Possible CAA	37 (19.5)
cSS (%)	188/190(99)
0	18 (9.6)
1 (focal)	55 (29.3)
2 (disseminated)	115 (61.2)
CMB binary	125/188 (66.5)

*AF* atrial fibrillation, *CAA* cerebral amyloid angiopathy, *CMB* cerebral microbleeds, *cSAH* cortical subarachnoid hemorrhage *cSS* cortical superficial siderosis, *DM* diabetes mellitus, *FU* follow-up, *HTN* hypertension, *ICH* intracerebral hemorrhage, *OAC* oral anticoagulation, *PMH* past medical history, *preICH* previous intracerebral hemorrhage, *preIS* previous ischemic stroke, *SD* standard deviation

^a^In case of missing values in the predictors the number is displayed as a fraction and percentage is based on complete cases

### Risk of outcome events

**Fig. 1:**
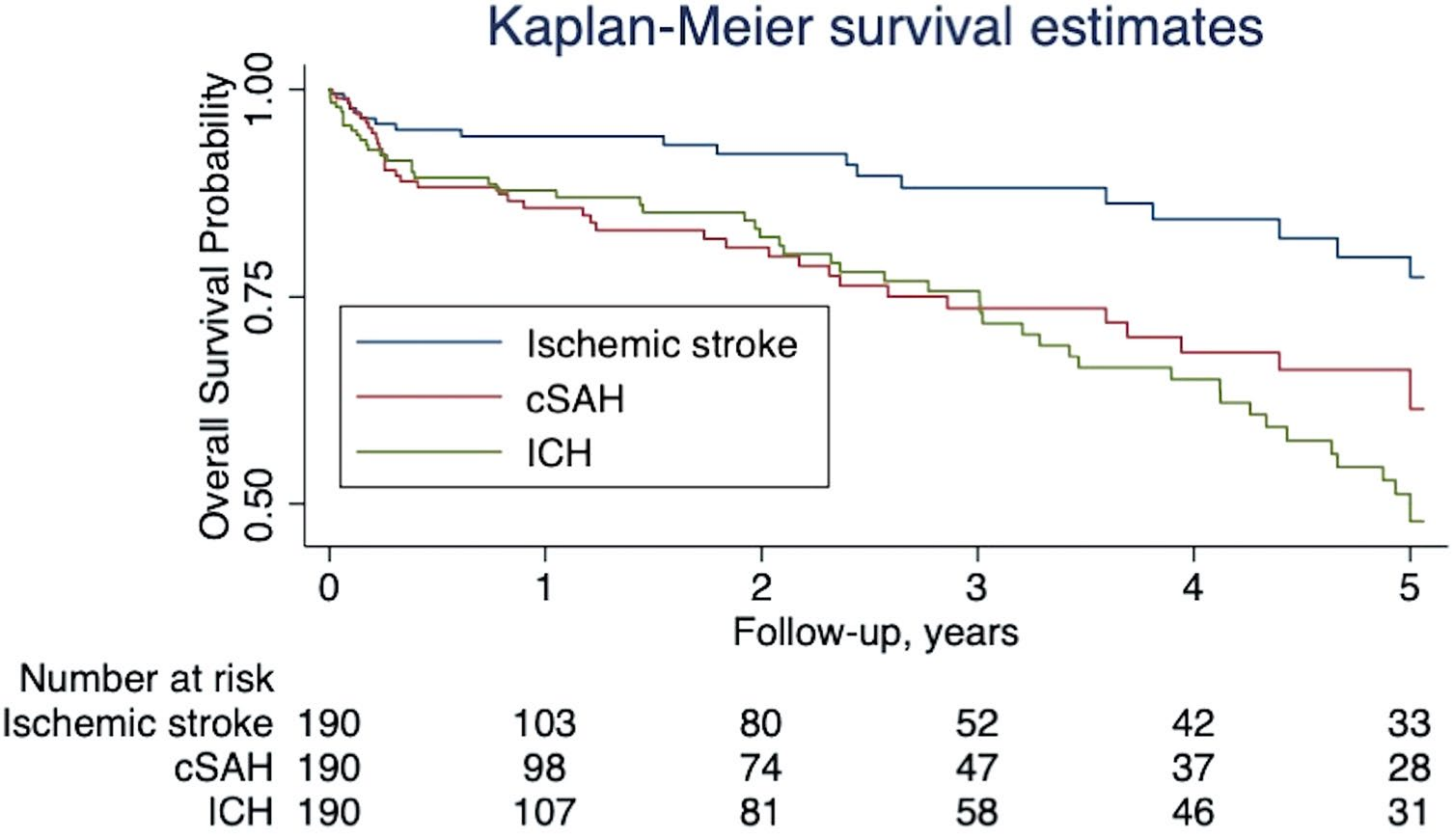
Kaplan Maier survival estimates for all outcome events in the whole cohort

Over the full period of follow-up, the frequency of events was as follows: 51 (26.8%) had an ICH, 39 (20.5%) a recurrent cSAH, and 19 (10%) an ischemic stroke; 31 (16.3%) died. Figure 1 demonstrates the event rate over time adjusting for censoring. Most outcome events occurred in participants with probable CAA; the proportions of patients fulfilling these criteria were: 50/51 (98%) patients with ICH; 35/39 (89.7%) patients with recurrent cSAH, and 15/19 (79%) with ischemic stroke.

#### Risk of symptomatic ICH during follow-up

**Fig. 2:**
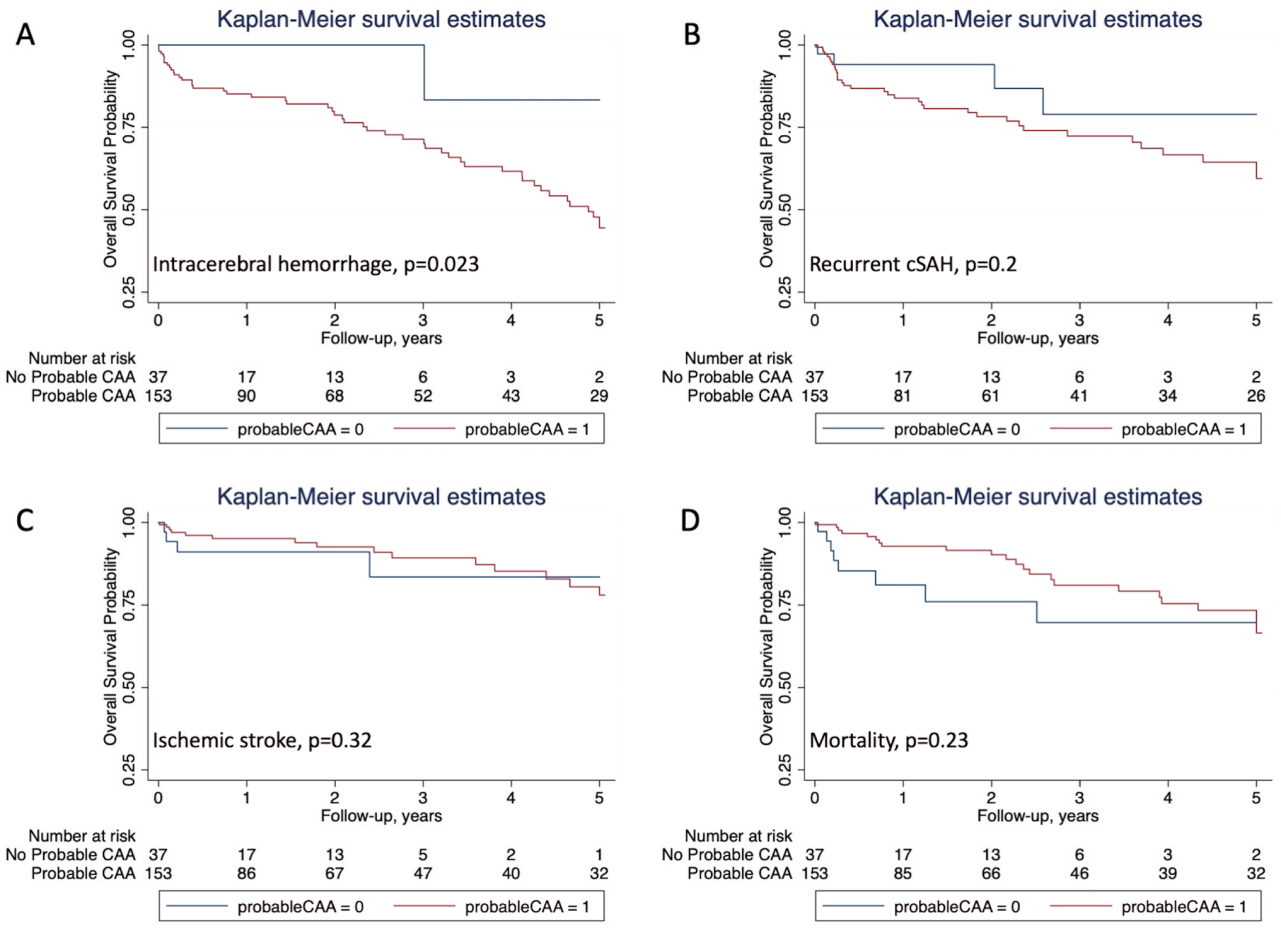
Kaplan Maier survival estimates based on outcome and probable CAA status: **A** ICH; **B** recurrent cSAH; **C** ischemic stroke; and **D** death

We observed 51 ICH over 385 patient-years, an absolute event rate of 13.2% (95% CI 9.9–17.4) per patient-year; the median time to ICH was 1.4 years (IQR 3.4), while 21.6% occurred in the first month (Fig. 1). The ICH rate for patients with probable CAA was 15.2% (95% CI 11.3–20) per patient-year, compared to 1.8% (95% CI 0.1–9.9) for those without probable CAA (*p *= 0.023; Table 2); see Fig. 2A for the Kaplan Meier (KM) survival estimates according to probable CAA diagnosis. Multivariable Cox regression, including the pre-specified variables probable CAA and age as well as anticoagulation, confirmed that patients with probable CAA had a higher risk of ICH than those without (HR 8.45, 95% CI 1.13–75.5, *p *= 0.02; Table 2). Despite the small HR, we did not find any evidence of association of OAC with ICH (HR 0.21; 95% CI 0.04–2.06).

**Table 2:** Predictors of ICH during follow-up for the full cohort

	No ICH on FU *N *= 139	ICH on FU *N *= 51	Univariable analysis			Multivariable analysis		
			HR	95% CI	*p* value	HR	95% CI	*p* value
Age, mean (SD), *N *(%)	74.4 (9.2)	75 (7.5)	1	0.96–1.04	0.96	1.01	0.97–1.05	0.76
Female sex, *N *(%)	62 (44.6)	24 (47.1)	1.14	0.65–1.99	0.66			
Current smoker	12/122^a^ (9.8)	5/47 (10.6)	1.69	0.64–4.5	0.29			
Current drinker	16/109 (14.7)	6/40 (15)	1.85	0.73–4.69	0.2			
PMH								
HTN, *N *(%)	83 (59.7)	34 (66.7)	1.08	0.6–1.96	0.8			
Hypercholesterolemia, *N *(%)	59 (42.5)	26 (51)	0.99	0.55–1.76	0.96			
DM, *N *(%)	17 (12.2)	9 (17.8)	1.1	0.51–2.36	0.82			
AF, *N *(%)	15/135 (11.1)	3 (5.9)	0.57	0.17–1.9	0.36			
OAC use, *N *(%)	14/135 (10.4)	1/50 (2)	0.25	0.03–1.85	0.17	0.21	0.04–2.06	0.21
Antiplatelet use, *N *(%)	46/135 (34.1)	17/50 (34)	0.84	0.44–1.59	0.59			
Statin use, *N* (%)	48/135 (35.6)	18/50 (36)	0.84	0.46–1.51	0.55			
Anti HTN use, *N *(%)	74/135 (54.8)	30/50 (60)	1.09	0.61–1.96	0.77			
Previous ICH, *N *(%)	16 (11.5)	8 (15.7)	1.03	0.46–2.29	0.95			
Previous IS, *N *(%)	17 (12.3)	4 (7.8)	0.6	0.21–1.7	0.34			
Restart antiplatelet, *N *(%)	38/122 (31.2)	16 (32.7)	0.87	0.46–1.66	0.68			
Restart OAC, *N *(%)	9/120 (7.5)	4 (8.2)	0.86	0.3–2.49	0.79			
Probable CAA, *N *(%)	103 (74.1)	50 (98)	10.26	1.37–76.77	**0.023**	8.45	1.13–75.5	**0.02**
cSS, *N *(%)	123/138 (89.1)	47/50 (94)	1.37	0.41–4.63	0.61			
CMB, *N *(%)	85/138 (61.6)	40/49 (81.6)	1.32	0.6–2.9	0.49			
WMH, *N *(%)	87/128 (68)	33/48 (68.8)	0.88	0.44–1.77	0.73			

Significant *p* values are marked in bold

*AF* atrial fibrillation, *CAA* cerebral amyloid angiopathy, *CI* confidence interval, *CMB* cerebral microbleeds, *cSS* cortical superficial siderosis, *DM* diabetes mellitus, *DWI* diffused white matter, *FU* follow-up, *HR* Hazard Ratio, *HTN* hypertension, *ICH* intracerebral hemorrhage, *OAC* oral anticoagulation, *PMH* past medical history, *preICH* previous intracerebral hemorrhage, *preIS* previous ischemic stroke, *SD* standard deviation, *WMH* white matter hyperdensity

^a^In case of missing values in the predictors the number is displayed as a fraction and percentage is based on complete cases

#### Risk of recurrent cSAH during follow-up

We observed 39 recurrent cSAH over 351 patient-years, an absolute event rate per patient-year of 11.1% (95% CI 7.9–15.2). The median time to recurrent cSAH was 1.3 years (IQR 3.3). The event rate was 11.9% (95% CI 8.3–16.5) for probable CAA and 7.1% (95% CI 1.9–18.3) for possible CAA (*p *= 0.2; Table 3). See Fig. 2B for the Kaplan Meier (KM) survival estimates according to probable CAA diagnosis. Patients with recurrent cSAH were more often male, and more often had probable CAA (Table 3). In the multivariable Cox regression model, adjusted for the prespecified variables probable CAA, age, sex and previous ischemic stroke and anticoagulation (Table 3), there was a higher risk of recurrent cSAH for probable CAA patients, but this was not significant at the 5% level (HR 3.66, 95% CI 0.84–15.9, *p *= 0.08). OAC use was not associated with the risk of recurrent cSAH (HR 1.65, 95% CI 0.51–5.35).

**Table 3:** Predictors of recurrent cSAH during follow-up

	No recSAH FU *N *= 151	recSAH FU *N *= 39	Univariable analysis			Multivariable analysis		
			HR	95% CI	*p* value	HR	95% CI	*p* value
Age, mean (SD)	74.7 (9.2)	73.9 (7.1)	0.97	0.94–1.01	0.19	0.98	0.94–1.02	0.24
Female sex, *N *(%)	74 (49)	12 (30.8)	0.47	0.23–0.95	0.04	0.42	0.19–0.9	0.03
Smoker	15/134^a^ (11.2)	2/35 (5.7)	0.57	0.13–2.47	0.45			
Drinker	19/116 (16.4)	3/3 (9.1)	0.6	0.18–2.04	0.41			
PMH								
HTN, *N *(%)	92 (60.9)	25 (64.1)	0.94	0.48–1.86	0.86			
Hypercholesterolemia, *N *(%)	67 (44.4)	18 (46.2)	1.3	0.67–2.52	0.44			
DM, *N *(%)	18 (11.9)	8 (20.5)	1.67	0.74–3.78	0.22			
AF, *N *(%)	13/149 (8.7)	5/37 (13.5)	1.66	0.61–453	0.32			
OAC use, *N *(%)	11/148 (7.4)	4/37 (10.8)	1.87	0.62–5.61	0.26	1.65	0.51–5.35	0.41
Antiplatelet use, *N *(%)	50/48 (33.8)	13/37 (35.1)	0.81	0.39–1.65	0.56			
Statin use, *N *(%)	52/148 (35.1)	14/37 (37.8)	1.18	0.59–2.36	0.63			
Anti HTN use, *N *(%)	84/148 (56.8)	20/37 (54.1)	0.79	0.39–1.56	0.49			
Previous ICH, *N *(%)	20 (13.3)	4 (10.3)	0.81	0.28–2.38	0.71			
Previous IS, *N *(%)	19/150 (12.7)	2 (5.1)	0.29	0.07–1.25	0.1	0.26	0.06–1.14	0.08
Restart antiplatelet, *N *(%)	43/134 (32.1)	11/37 (29.7)	0.89	0.42–1.91	0.89			
Restart OAC, *N *(%)	12/133 (7.5)	3/36 (8.3)	0.92	0.27–3.15	0.92			
Probable CAA, *N *(%)	118 (78.2)	35 (89.7)	2.07	0.69–6.23	0.2	3.66	0.84–15.9	0.08
cSS, *N *(%)	132/149 (88.6)	38 (97.4)	5.05	0.67–38.07	0.12			
CMB, *N *(%)	96/148 (64.9)	29 (74.4)	1.2	0.54–2.65	0.65			
WMH, *N *(%)	94/137 (68.6)	26 (66.7)	0.67	0.31–1.44	0.3			

*AF* atrial fibrillation, *CAA* cerebral amyloid angiopathy, *CI* confidence interval, *CMB* cerebral microbleeds, *recSAH* recurrent cortical subarachnoid hemorrhage, *cSS* cortical superficial siderosis, *DM* diabetes mellitus, *DWI* diffused white matter, *FU* follow-up, *HR* Hazard Ratio, *HTN* hypertension, *OAC* oral anticoagulation, *PMH* past medical history, *preICH* previous intracerebral hemorrhage, *preIS* previous ischemic stroke, *SD* standard deviation, *WMH* white matter hyperdensity

^a^In case of missing values in the predictors the number is displayed as a fraction and percentage is based on complete cases

#### Risk of any intracranial haemorrhage during follow-up

The risk of any intracranial haemorrhage (recurrent ICH, cSAH, or both) per patient-year was 21.4% (95% CI 16.7–26.9); 17 patients had both recurrent ICH and cSAH.

#### Risk of ischemic stroke on follow-up

We observed 19 symptomatic ischemic stroke events over 373 patients-years, an absolute event rate per patient-year of 5.1% (95% CI 3.1–8). The median time to ischemic stroke was 1.3 years (IQR 3.3). The event rate for patients with probable CAA was 4.7% (95% CI 2.6–7.7) and for patients with possible CAA 7.5% (95% CI 2–19.2; *p *= 0.32; Table 4, Fig. 2C). Patients with ischemic stroke on follow-up were more often male, more frequently had a history of hypertension, were on anticoagulation, and had probable CAA (Table 4). In our multivariable model, adjusted with the pre-specified variables age and anticoagulation (Table 4), probable CAA was not associated with a higher risk of ischemic stroke (HR 0.56, 95% CI 0.17–1.82, *p *= 0.33).

**Table 4:** Predictors of ischemic stroke during follow-up

	No IS on FU *N *= 171	IS on FU *N *= 19	Univariable analysis			Multivariable analysis		
			HR	95% CI	*p* value	HR	95% CI	*p* value
Age, mean (SD)	74.5 (9)	74.6 (6.5)	1.01	0.95–1.07	0.8	1	0.94–1.07	0.9
Female sex, *N *(%)	79 (46.2)	7 (36.8)	0.72	0.28–1.85	0.5			
Smoker	15/150^a^ (10)	2 (10.5)	1.25	0.28–5.56	0.77			
Drinker	19/131 (14.5)	3/18 (16.7)	1.83	0.5–6.7	0.36			
PMH								
HTN, *N *(%)	103 (60.2)	14 (73.7)	1.71	0.61–4.81	0.31			
Hypercholesterolemia, *N *(%)	78 (45.6)	7 (36.8)	0.71	0.27–1.88	0.49			
DM, *N *(%)	22 (12.9)	4 (21.1)	1.71	0.55–5.34	0.36			
AF, *N *(%)	15/2167 (9)	3 (15.8)	2	0.55–7.33	0.3			
OAC use, *N *(%)	11/166 (6.6)	4 (21.1)	3.63	1.13–11.68	0.03	3.44	1.07–11.06	0.04
Antiplatelet use, *N *(%)	56/166 (33.7)	7 (36.8)	1.06	0.4–2.77	0.91			
Statin use, *N *(%)	60/166 (36.1)	6 (31.6)	0.86	0.32–2.32	0.77			
Anti HTN use, *N *(%)	93/166 (56)	11 (5791)	1.02	0.4–2.6	0.97			
Previous ICH, *N *(%)	23 (13.5)	1 (5.3)	0.36	0.05–2.71	0.32			
Previous IS, *N *(%)	18/170 (10.6)	3 (15.8)	1.75	0.49–6.21	0.39			
Restart antiplatelet, *N *(%)	48/152 (31.6)	6 (31.6)	1.14	0.42–3.13	0.79			
Restart OAC, *N *(%)	12/150 (8)	1 (5.3)	0.53	0.07–4.07	0.54			
Probable CAA, *N *(%)	138 (80.7)	15 (78.9)	0.55	0.16–1.81	0.32	0.56	0.17–1.82	0.33
cSS, *N *(%)	155/169 (91.7)	15 (79)	0.39	0.12–1.24	0.11			
CMB, *N *(%)	110/168 (65.5)	15 (79)	1.15	0.36–3.67	0.82			
WMH, *N *(%)	107/157 (68.2)	13 (68.4)	0.73	0.26–2.06	0.56			

*AF* atrial fibrillation, *CAA* cerebral amyloid angiopathy, *CI* confidence interval, *CMB* cerebral microbleeds, *cSS* cortical superficial siderosis, *DM* diabetes mellitus, *DWI* diffused white matter, *FU* follow-up, *HR* Hazard Ratio, *HTN* hypertension, *OAC* oral anticoagulation, *PMH* past medical history, *preICH* previous intracerebral hemorrhage, *preIS* previous ischemic stroke, *SD* standard deviation, *WMH* white matter hyperdensity

^a^In case of missing values in the predictors the number is displayed as a fraction and percentage is based on complete cases

#### Risk of death during follow-up

We observed 31 deaths over 373 patient-years, an overall absolute event rate of 8.3% (95%CI 5.6–11.8) per patient-year. The median time to death was 1.2 years (IQR 3.3). There was no statistically significant difference in mortality between patients with probable CAA [7.3% for patients with probable CAA (95% CI 4.6–10.9)] and those without [14.2% for patients without probable CAA (95% CI 6.1–28); *p *= 0.23; Table 5, Fig. 2D]. Patients who died were older, and more often had hypertension, and were on antihypertensive medication more often (Table 5). In our multivariable model, adjusted for the pre-specified variables age and hypertension (Table 5), probable CAA was not associated with death (HR 0.54, 95% CI 0.16–1.78, *p *= 0.31). In a sensitivity analysis, adding smoking status to the multivariable model did not change the findings (results not shown).

**Table 5:** Predictors of mortality during follow-up

	Survived *N *= 159	Death *N *= 31	Univariable analysis			Multivariable analysis		
			HR	95% CI	*p* value	HR	95% CI	*p* value
Age, mean (SD)	74 (9)	77.5 (6.7)	1.04	0.99–1.1	0.14	1.04	0.98–1.11	0.21
Female sex, *N *(%)	74 (46.5)	12 (38.7)	0.89	0.43–1.87	0.76			
Smoker	12/139^a^ (8.6)	5/30 (16.7)	2.47	0.88–6.9	0.09			
Drinker	18/125 (14.4)	4/24 (16.7)	1.68	0.54–5.25	0.37			
PMH								
HTN, *N *(%)	93 (58.5)	24 (77.4)	1.82	0.77–4.27	0.17	2.27	1.24–4.17	0.008
Hypercholesterolemia, *N *(%)	70 (44)	15 (48.4)	1.06	0.51–2.24	0.87			
DM, *N *(%)	20 (12.6)	6 (19.4)	1.3	0.51–3.31	0.58			
AF, *N *(%)	13/155 (8.4)	5 (16.1)	1.54	0.56–4.25	0.41			
OAC use, *N *(%)	12/155 (7.7)	3/30 (10)	1.26	0.36–4.42	0.71			
Antiplatelet use, *N *(%)	55/155 (35.5)	8/30 (26.7)	0.66	0.28–1.55	0.34			
Statin use, *N *(%)	56/155 (36.1)	10/30 (33.3)	0.8	0.36–1.75	0.57			
Anti HTN, *N *(%)	83/155 (53.6)	21/30 (70)	1.57	0.7–3.53	0.28			
Previous ICH, *N *(%)	20 (12.6)	4 (12.9)	0.96	0.32–2.85	0.94			
sPrevious IS, *N *(%)	16 (10.1)	5 (16.1)	1.17	0.43–3.15	0.76			
Restart antiplatelet, *N *(%)	46/140 (32.9)	8 (25.8)	0.76	0.32–1.77	0.52			
Restart OAC, *N *(%)	9/138 (6.5)	4 (12.9)	1.2	0.4–3.56	0.75			
Probable CAA, *N *(%)	130 (81.8)	23 (74.2)	0.57	0.23–1.42	0.23	0.54	0.16–1.78	0.31
cSS, *N *(%)	146/158 (92.4)	24/30 (80)	0.31	0.12–0.81	**0.02**			
CMB, *N* (%)	104/158 (65.8)	21/29 (72.4)	0.94	0.38–2.33	0.63			
WMH, *N* (%)	100/146 (68.5)	20/30 (66.7)	0.6	0.25–1.43	0.25			

Significant *p* values is marked in bold

*AF* atrial fibrillation, *CAA* cerebral amyloid angiopathy, *CI* confidence interval, *CMB* cerebral microbleeds, *cSS* cortical superficial siderosis, *DM* diabetes mellitus, *DWI* diffused white matter, *FU* follow-up, *HR* Hazard Ratio, *HTN* hypertension, *OAC* oral anticoagulation, *PMH* past medical history, *preICH* previous intracerebral hemorrhage, *preIS* previous ischemic stroke, *SD* standard deviation, *WMH* white matter hyperdensity

^a^In case of missing values in the predictors the number is displayed as a fraction and percentage is based on complete cases

When comparing patients with ICH versus ischemic stroke on follow-up, patients suffering an ICH had a higher mortality rate than those who had an ischemic stroke (45.5% vs 20.5%). The proportional hazard assumption was not violated for any of the outcomes. As a sensitivity analysis, we conducted a competing risk analysis for all of the outcome events, which demonstrated similar results (results not shown).

#### Influence of symptoms at presentation on outcome events

In a final step, we adjusted for symptoms on presentation (positive, negative or spreading symptoms) in the univariable and multivariable model; clinical presentation did not influence the risks of stroke or death during follow-up (results not shown).

#### Influence of age on outcome events

In a post hoc analysis we included an age term dichotomized at age 70 in the Cox models (based on a previous publication which used this cutoff) [15]; there were no significant differences between age > 70 and < 70 in any of our outcomes of interest (data not shown).

#### Use of antithrombotic drugs during follow-up

Data on starting or restarting antiplatelets and oral anticoagulants were available for 171/190 (90%) and 169/190 (89%) patients, respectively; 54/171 (31.6%) were started or restarted on antiplatelets and 13/169 (7.7%) on oral anticoagulants. Neither antiplatelet nor anticoagulant use after cSAH were associated with any of the outcome events.

## Discussion

Our pooled individual patient-data analysis confirms that patients who had a cSAH due to suspected CAA have a high rate of future intracranial haemorrhage (both ICH and cSAH), with an overall intracranial haemorrhage risk (ICH, cSAH, or both) of 21.4% per patient-year. The rates were highest within the first month after cSAH, during which 21.6% of ICH and 12.8% of cSAH occurred. The rate of ischemic stroke was much lower at 5.1% per patient-year, while mortality was 8.3% per patient-year. In adjusted analyses, we found that the rate of future ICH was around 8.5 times higher in patients fulfilling the modified Boston criteria for probable CAA compared to those without probable CAA.

Our study expands on previous smaller studies reporting the rates of ICH and recurrent cSAH in patients presenting with cSAH and emphasizes that presentation with cSAH can be a sentinel event warning of impending ICH. Due to larger patient numbers and individual patient-data, we provide more precise estimates of the high future ICH risk in cSAH patients fulfilling the modified Boston criteria for probable and possible CAA [31, 32]; we found that the ICH rate for patients with probable CAA was 15.2% (95% CI 11.3–20) per patient-year, compared to 1.8% (95% CI 0.001–9.9) for those without (*p *= 0.023). Patients fulfilling the modified Boston criteria for probable CAA had a higher rate of recurrent cSAH on follow-up, but this finding was not statistically significant.

The high rates of ICH and recurrent cSAH are also consistent with another previous study of 38 patients with cSAH, over a mean of 24 months of follow-up, during which 15 (39%) experienced recurrent cSAHs and 14 (37%) suffered lobar ICHs; notably, of 22 new ICHs, 17 occurred at sites of previous cSAHs or cSS [14]. Moreover, in four patients, imaging demonstrated early meningeal enhancement, suggesting active vascular leakage associated with leptomeningeal CAA, and rapid expansion of cSAH into the parenchyma causing an ICH [19]. These data, together with our study, are consistent with the idea that cSAH—presumably associated with severe leptomeningeal CAA—could in some instances be an initial step in ICH formation.

Our study also provides new information on the future rate of ischemic stroke after cSAH [12], confirming that the risk of ischemic stroke is much lower than that of recurrent ICH or cSAH.

Such information on hemorrhagic and ischemic cerebral events is important for informing management decisions, particularly regarding antithrombotic therapy. Indeed, we found that 39.5% of our cohort were taking antithrombotic drugs at the time of the cSAH event, suggesting that whether to restart such drugs is a common clinical dilemma after cSAH. In our study, only 31.6% were started or restarted on antiplatelets and 7.7% on anticoagulation, suggesting there is anxiety about future intracranial bleeding risk in clinical practice. Although antithrombotic therapy (antiplatelets or anticoagulants) could reduce the risk of ischemic events, they might increase the risk of future ICH or cSAH. Our study did not find evidence that restarting antithrombotic therapy after cSAH increases the rate of intracranial bleeding, but this observation is likely to be affected by bias and confounding including physician decisions to restart these agents.

Despite the much higher risks of ICH and cSAH in people with probable vs possible CAA, we did not find evidence of a higher mortality rate in this group, although the rate in our cohort was comparable to previous reports [33].

We found that patients with cSAH attributed to probable or possible CAA have a much higher annual risk of future intracranial haemorrhage (ICH or cSAH) than ischemic stroke [13.2% (95% CI 9.9–17.4) and 11.1% (95%CI 7.9–15.2), respectively, compared with a 5.1% (95% CI 3.1–8)]. The substantially higher ICH risk in probable compared to possible CAA (15.2% vs 1.8% per year)—with a similar ischemic stroke risk regardless of CAA status—might favor avoiding antithrombotics for patients with cSAH who meet the modified Boston criteria for probable CAA. There are no other observational data or randomized controlled trials addressing the risks of (re)starting antithrombotic drugs after cSAH. Data on whether antiplatelet drugs affect recurrent ICH risk after a symptomatic ICH are conflicting: while two small single center studies gave inconsistent data on whether the rate of recurrent ICH was increased by the use of aspirin [34, 35], the RESTART randomized trial in ICH survivors found no evidence of an increase in the risk of recurrent intracerebral haemorrhage with antiplatelet therapy for patients on antithrombotic therapy for the prevention of occlusive vascular disease when they developed ICH [36]. However, this trial included small numbers of patients with disseminated superficial siderosis. We found a high risk of ICH after cSAH despite most patients not receiving antithrombotic therapy. Thus, in those judged to have a low vaso-occlusive risk starting or restarting them might be best avoided. Our finding that about one in five ICH occur in the first month raises particular concern for antithrombotic therapy during this early period following cSAH. Although randomized data are needed to inform antithrombotic decisions after cSAH, the rarity of cSAH and lack of clinician equipoise might make such trials challenging.

Our pooled analysis approach has several strengths. We have included the largest number of patients with cSAH associated with probable or possible CAA studied to date, with a long follow-up period, allowing us to provide more precise estimates of the rates of future ICH, cSAH, as well as ischemic stroke after a previous cSAH, including data on the longer-term time course of these events. The inclusion of detailed individual patient data additionally allowed us to evaluate multivariable models. Reassuringly, in our pooled analysis, the risk of bias was low, so any loss to follow-up is most likely due to random (non-informative) censoring.

However, our study also has limitations. This was a retrospective study, although we only included studies where investigators had systematically and prospectively collected data on the follow-up events of interest as defined in the protocol. Additionally, imaging was not reviewed centrally. This is a potential source of bias as agreement between the different centers could not be tested. However, uniform definitions of CAA were applied by trained raters as per our predefined study protocol. Also, our rate of spreading symptoms was surprisingly low which we think to be due to the retrospective nature of the study and reflects an underestimation. This needs to be verified in an independent large cohort.

## Conclusions

We confirm that patients with cSAH are at high risk of future ICH and recurrent cSAH (13.2% and 11.1% per patient-year, respectively), with a high early risk. By contrast, the risk of ischemic stroke is much lower (5.1% per patient-year). Probable vs possible CAA is a predictor of higher ICH but not ischemic stroke risk. Our data provide precise risk estimates of key vascular outcomes after cSAH that can help inform prognosis and management decisions after cSAH.

## Supplementary Information

Below is the link to the electronic supplementary material.

Supplementary file1 (DOCX 117 KB)

Supplementary file2 (PDF 961 KB)

## Data Availability

Anonymized data will be shared on request from any qualified investigator, subject to approval of the participating collaborators.

## References

[1] Beitzke M, Gattringer T, Enzinger C, Wagner G, Niederkorn K, Fazekas F (2011). Clinical presentation, etiology, and long-term prognosis in patients with nontraumatic convexal subarachnoid haemorrhage. Stroke; J Cereb Circ.

[2] Hostettler IC, Werring DJ (2018). Acute convexity subarachnoid haemorrhage: what the neurosurgeon needs to know. World Neurosurg.

[3] Ni J, Auriel E, Jindal J, Ayres A, Schwab KM, Martinez-Ramirez S, Gurol EM, Greenberg SM, Viswanathan A (2015). The characteristics of superficial siderosis and convexity subarachnoid haemorrhage and clinical relevance in suspected cerebral amyloid angiopathy. Cerebrovasc Dis.

[4] Kumar S, Goddeau RP, Selim MH, Thomas A, Schlaug G, Alhazzani A, Searls DE, Caplan LR (2010). Atraumatic convexal subarachnoid haemorrhage: clinical presentation, imaging patterns, and etiologies. Neurology.

[5] Stanton JED, Chandratheva A, Wilson D, Hostettler IC, Islam S, Werring DJ (2020). Clinical features distinguish cerebral amyloid angiopathy-associated convexity subarachnoid haemorrhage from suspected TIA. J Neurol.

[6] Raposo N, Viguier A, Cuvinciuc V, Calviere L, Cognard C, Bonneville F, Larrue V (2011). Cortical subarachnoid haemorrhage in the elderly: a recurrent event probably related to cerebral amyloid angiopathy. Eur J Neurol.

[7] Vinters HV (1987). Cerebral amyloid angiopathy. A critical review. Stroke; J Cereb Circ.

[8] Yamada M (2000). Cerebral amyloid angiopathy: an overview. Neuropathology.

[9] Charidimou A, Gang Q, Werring DJ (2012). Sporadic cerebral amyloid angiopathy revisited: recent insights into pathophysiology and clinical spectrum. J Neurol Neurosurg Psychiatry.

[10] Viswanathan A, Greenberg SM (2011). Cerebral amyloid angiopathy in the elderly. Ann Neurol.

[11] Wilson D, Hostettler IC, Ambler G, Banerjee G, Jager HR, Werring DJ (2017). Convexity subarachnoid haemorrhage has a high risk of intracerebral haemorrhage in suspected cerebral amyloid angiopathy. J Neurol.

[12] Wilson D, Hostettler IC, Ambler G, Banerjee G, Jager HR, Werring DJ (2017). Convexity subarachnoid haemorrhage has a high risk of intracerebral haemorrhage in suspected cerebral amyloid angiopathy. J Neurol.

[13] Apoil M, Cogez J, Dubuc L, Bataille M, de la Sayette V, Touze E, Viader F (2013). Focal cortical subarachnoid haemorrhage revealed by recurrent paresthesias: a clinico-radiological syndrome strongly associated with cerebral amyloid angiopathy. Cerebrovasc Dis.

[14] Beitzke M, Enzinger C, Wunsch G, Asslaber M, Gattringer T, Fazekas F (2015). Contribution of convexal subarachnoid haemorrhage to disease progression in cerebral amyloid angiopathy. Stroke; J Cereb Circ.

[15] Bruno VA, Lereis VP, Hawkes M, Ameriso SF (2013). Nontraumatic subarachnoid haemorrhage of the convexity. Curr Neurol Neurosci Rep.

[16] Geraldes R, Sousa PR, Fonseca AC, Falcao F, Canhao P, e Canhao P (2014). Nontraumatic convexity subarachnoid haemorrhage: different etiologies and outcomes. J Stroke Cerebrovasc Dis: Off J Natl Stroke Assoc.

[17] Graff-Radford J, Fugate JE, Klaas J, Flemming KD, Brown RD, Rabinstein AA (2016). Distinguishing clinical and radiological features of non-traumatic convexal subarachnoid haemorrhage. Eur J Neurol.

[18] Khurram A, Kleinig T, Leyden J (2014). Clinical associations and causes of convexity subarachnoid haemorrhage. Stroke; J Cereb Circ.

[19] Ly JV, Singhal S, Rowe CC, Kempster P, Bower S, Phan TG (2015). Convexity subarachnoid haemorrhage with PiB positive pet scans: clinical features and prognosis. J Neuroimaging: Off J Am Soc Neuroimaging.

[20] Martinez-Lizana E, Carmona-Iragui M, Alcolea D, Gomez-Choco M, Vilaplana E, Sanchez-Saudinos MB, Clarimon J, Hernandez-Guillamon M, Munuera J, Gelpi E, Gomez-Anson B, de Juan-Delago M, Delgado-Mederos R, Montaner J, Ois A, Amaro S, Blesa R, Marti-Fabregas J, Lleo A, Fortea J (2015). Cerebral amyloid angiopathy-related atraumatic convexal subarachnoid haemorrhage: an ARIA before the tsunami. J Cereb Blood flow and metab: Off J Int Soc Cereb Blood Flow Metab.

[21] Mas J, Bouly S, Mourand I, Renard D, de Champfleur N, Labauge P (2013). Focal convexal subarachnoid haemorrhage: clinical presentation, imaging patterns and etiologic findings in 23 patients. Revue neurologique.

[22] Refai D, Botros JA, Strom RG, Derdeyn CP, Sharma A, Zipfel GJ (2008). Spontaneous isolated convexity subarachnoid haemorrhage: presentation, radiological findings, differential diagnosis, and clinical course. J Neurosurg.

[23] Linn J, Halpin A, Demaerel P, Ruhland J, Giese AD, Dichgans M, van Buchem MA, Bruckmann H, Greenberg SM (2010). Prevalence of superficial siderosis in patients with cerebral amyloid angiopathy. Neurology.

[24] Ducros A, Boukobza M, Porcher R, Sarov M, Valade D, Bousser MG (2007). The clinical and radiological spectrum of reversible cerebral vasoconstriction syndrome. A prospective series of 67 patients. Brain: J Neurology.

[25] Moher D, Liberati A, Tetzlaff J, Altman DG, Group P (2009). Preferred reporting items for systematic reviews and meta-analyses: the PRISMA statement. BMJ.

[26] von Elm E, Altman DG, Egger M, Pocock SJ, Gotzsche PC, Vandenbroucke JP, Initiative S (2007). The strengthening the reporting of observational studies in epidemiology (STROBE) statement: guidelines for reporting observational studies. Lancet.

[27] van Swieten JC, Hijdra A, Koudstaal PJ, van Gijn J (1990). Grading white matter lesions on CT and MRI: a simple scale. J Neurol Neurosurg Psychiatry.

[28] Gregoire SM, Chaudhary UJ, Brown MM, Yousry TA, Kallis C, Jager HR, Werring DJ (2009). The microbleed anatomical rating scale (MARS): reliability of a tool to map brain microbleeds. Neurology.

[29] Charidimou A, Linn J, Vernooij MW, Opherk C, Akoudad S, Baron JC, Greenberg SM, Jager HR, Werring DJ (2015). Cortical superficial siderosis: detection and clinical significance in cerebral amyloid angiopathy and related conditions. Brain: J Neurol.

[30] Haacke EM, Xu Y, Cheng YC, Reichenbach JR (2004). Susceptibility weighted imaging (SWI). Magn Reson Med.

[31] Greenberg SM, Eng JA, Ning M, Smith EE, Rosand J (2004). Haemorrhage burden predicts recurrent intracerebral haemorrhage after lobar haemorrhage. Stroke; J Cereb Circ.

[32] van Etten ES, Auriel E, Haley KE, Ayres AM, Vashkevich A, Schwab KM, Rosand J, Viswanathan A, Greenberg SM, Gurol ME (2014). Incidence of symptomatic haemorrhage in patients with lobar microbleeds. Stroke; J Cereb Circ.

[33] Calviere L, Viguier A, Patsoura S, Rousseau V, Albucher JF, Planton M, Pariente J, Cognard C, Olivot JM, Bonneville F, Raposo N (2019). Risk of intracerebral haemorrhage and mortality after convexity subarachnoid haemorrhage in cerebral amyloid angiopathy. Stroke; J Cereb Circ.

[34] Biffi A, Halpin A, Towfighi A, Gilson A, Busl K, Rost N, Smith EE, Greenberg MS, Rosand J, Viswanathan A (2010). Aspirin and recurrent intracerebral haemorrhage in cerebral amyloid angiopathy. Neurology.

[35] Viswanathan A, Rakich SM, Engel C, Snider R, Rosand J, Greenberg SM, Smith EE (2006). Antiplatelet use after intracerebral haemorrhage. Neurology.

[36] Collaboration R (2019). Effects of antiplatelet therapy after stroke due to intracerebral haemorrhage (RESTART): a randomised, open-label trial. Lancet.

