# Supplementary material for: Risk of intracranial haemorrhage and ischaemic stroke after convexity subarachnoid haemorrhage in cerebral amyloid angiopathy: international individual patient data pooled analysis
Source: J Neurol. 2021 Jul 17;269(3):1427–38. doi: 10.1007/s00415-021-10706-3 (PMC8857171; doi:10.1007/s00415-021-10706-3)
Supplement: Supplementary file 1 — Supplementary file1 (DOCX 117 KB) [file 415_2021_10706_MOESM1_ESM.docx]

**Risk of intracranial hemorrhage and ischemic stroke after convexity subarachnoid hemorrhage in cerebral amyloid angiopathy: international individual patient data pooled analysis**

Isabel Charlotte Hostettler MD^1^, Duncan Wilson PhD^1^, Catherine Arnold Fiebelkorn MD^2^, Diane Aum MD^3^, Sebastián Francisco Ameriso MD^4^, Federico Eberbach MD^4^, Markus Beitzke MD^5^, Timothy Kleinig PhD^6^, Thanh Phan FRACP PhD^7^, Sarah Marchina PhD^8^, Romain Schneckenburger MD^9^, Maria Carmona-Iragui MD^10^, Andreas Charidimou PhD^11^, Isabelle Mourand MD^12^, Sara Parreira MD^13^, Gareth Ambler PhD^14^, Hans Rolf Jäger MD^15^, Shaloo Singhal MD^7^, John Ly MD^7^, Henry Ma FRACP, PhD^7^, Emmanuel Touzé E PhD^16^, Ruth Geraldes MD^17, 18^, Ana Catarina Fonseca MD^13^, Teresa Melo MD^13^, Pierre Labauge MD^12^, Pierre-Henry Lefèvre MD^19^, Anand Viswanathan PhD^11^, Steven Mark Greenberg PhD^11^, Juan Fortea MD^10^, Marion Apoil MD^9^, Marion Boulanger MD^9^, Fausto Viader MD^9^, Sandeep Kumar MD^8^, Velandai Srikanth FRACP, PhD^7^, Ashan Khurram FRACP^6^, Franz Fazekas MD^5^, Veronica Bruno MD^4^, Gregory Joseph Zipfel MD^3^, Daniel Refai MD^20^, Alejandro Rabinstein MD^2^, Jonathan Graff-Radford MD^2^, David John Werring FRCP PhD^1^

*^1^ Stroke Research Centre, University College London, Institute of Neurology, London, UK*

*^2^Department of Neurology, Mayo Clinic, Rochester, MN, USA*

*^3^Department of Neurological Surgery, Washington University School of Medicine, St. Louis, MO, USA*

*^4^Institute for Neurological Research, Fleni, Buenos Aires, Argentina*

*^5^Department of Neurology, Medical University of Graz, Austria*

*^6^Department of Neurology, Royal Adelaide Hospital, Adelaide, Australia*

*^7^Department of Neurology, Monash Health and Stroke and Ageing Research Group, Department of Medicine, School of Clinical Sciences, Monash University, Melbourne, Australia*

*^8^Department of Neurology, Stroke Division, Beth Israel Deaconess Medical Center, Harvard Medical School, Boston, MA, USA*

*^9^Department of Neurology, CHU Caen Normandie, Caen, France*

*^10^Memory Unit, Department of Neurology, Hospital de la Santa Creu i Sant Pau, Institut Investigació Biomèdica Sant Pau, Universitat Autònoma de Barcelona, Barcelona, Spain*

*^11^J. Philip Kistler Stroke Research Center, Department of Neurology, Massachusetts General Hospital and Harvard Medical School, Boston, MA, USA*

*^12^Department of Neurology, CHU de Montpellier, Hôpital Gui-de-Chauliac, Montpellier, France*

*^13^Stroke Unit, Department of Neuroscience, Hospital de Santa Maria, Lisbon, Portugal*

*^14^Department of Statistical Science, UCL, London, WC1E 6BT, UK*

*^15^Neuroradiological Academic Unit, Department of Brain Repair & Rehabilitation, University College London, Institute of Neurology, London, UK*

*^16^* *Normandy University, UNICAEN, INSERM U1237, Caen, France*

*^17^Nuffield Department of Clinical Neurosciences, Oxford University Hospitals, Oxford, UK*

*^18^* *Neurology department, Frimley Health Foundation Trust*

*^19^Department of Neuroradiology, CHU de Montpellier, Hôpital Gui-de-Chauliac, Montpellier, France*

*^20^Department of Neurosurgery, Emory University, Atlanta, GA, USA*

**Corresponding author:** Professor David Werring, FRCP, PhD, National Hospital of Neurology and Neurosurgery, Institute of Neurology, University College London, Queen Square, WC1N London, United Kingdom, Phone: +44 20 3447 5994, Fax: +44 20 7833 8613, Email: d.werring@ucl.ac.uk

**Online only Supplementary Material**

TABLES

Table e-1. Quality Assessment (Method: Cochrane Collaboration’s Tool for Assessing Risk of Bias)

Table e-2. Patient characteristics of the included cohorts

Table e-3. Characteristics of patients according to probable CAA

FIGURE TITLES

Figure e-1. Prisma Study flow chart

**Table e-1. Quality Assessment (Method: Cochrane Collaboration’s Tool for Assessing Risk of Bias)**

|  | Selection of Cohorts | Assessment of Exposure | Outcomes of interest not present | Adjusting for variables associated with outcome | Confidence in Assessment of Outcome | Follow-up adequate? | Information on CAA (modified Boston criteria) not available |
| --- | --- | --- | --- | --- | --- | --- | --- |
| UCLH^1^ | ++ | ++ | ++ | ++ | ++ | ++ | ++ |
| CHU Caen^2^ | ++ | ++ | ++ | ++ | ++ | + | + |
| Klinikum Graz^3, 4^ | ++ | ++ | ++ | ++ | ++ | ++ | ++ |
| Lisbon^5^ | ++ | ++ | ++ | ++ | ++ | + | + |
| Mayo^6^ | ++ | ++ | ++ | ++ | ++ | + | ++ |
| CHU Montpellier^7^ | ++ | ++ | ++ | ++ | ++ | ++ | ++ |
| Sant Pau Barcelona^8^ | ++ | ++ | ++ | ++ | ++ | ++ | ++ |
| Royal Adelaide^9^ | ++ | ++ | ++ | ++ | ++ | + | ++ |
| FLENI Argentina^10^ | ++ | ++ | ++ | ++ | ++ | + | ++ |
| Beth Israel USA^11^ | ++ | ++ | ++ | ++ | ++ | ++ | ++ |
| Mass Gen USA^12^ | ++ | ++ | ++ | ++ | ++ | + | ++ |
| Monash University^13^ | ++ | ++ | ++ | ++ | ++ | ++ | ++ |
| Emory Clinic^14^ | ++ | ++ | ++ | ++ | ++ | + | + |

CAA=cerebral amyloid angiopathy

Four-point scale: ++ Low risk of bias, + minor risk of bias, -some risk of bias, -- high risk of bias

**Table e-2: patient characteristics of the included cohorts**

|  | **Study Centers** | | | | | | | | | | | | |
| --- | --- | --- | --- | --- | --- | --- | --- | --- | --- | --- | --- | --- | --- |
| **Variable** | **UCLH** | **CHU Caen** | **Klinikum Graz** | **Lisbon** | **Mayo** | **CHU Montpellier** | **Sant Pau Barcelona** | **Royal Adelaide** | **FLENI Argentina** | **Beth Israel** | **Mass Gen**  **USA** | **Monash University** | **Emory Clinic** |
|  | **N=18** | **N=12** | **N=28** | **N=3** | **N=22** | **N=9** | **N=20** | **N=12** | **N=12** | **N=9** | **N=13** | **N=25** | **N=7** |
| **Age, mean (SD)** | 69 (13.1) | 79 (7.7) | 74.3 (6.7) | 75.7 (9.5) | 73.4 (7.1) | 70.9 (5.5) | 80.5 (5.5) | 76.1 (6.3) | 71.7 (8.2) | 75.4 (8) | 77.3 (5.5) | 74.5 (10.6) | 68.3 (12) |
| **Probable CAA, N (%)** | 18 (100) | 11 (91.7) | 19 (67.9) | 3 (100) | 20 (90.0) | 9 (100) | 8 (40) | 11 (91.7) | 11 (91.7) | 8 (88.9) | 9 (69.2) | 23 (92) | 3 (42.9) |
| **Possible CAA, N (%)** | 0 | 1 (8.3) | 9 (32.1) | 0 | 2 (9.1) | 0 | 12 (60) | 1 (8.3) | 1 (8.3) | 1 (11.1) | 4 (30.8) | 2 (8) | 4 (57.1) |
| **Female Sex, N(%)** | 8 (44.4) | 5 (41.7) | 13 (46.4) | 0 | 12 (54.6) | 6 (66.7) | 8 (40) | 3 (25) | 6 (50) | 5 (55.6) | 5 (38.5) | 10 (40) | 5 (71.4) |
| **Current Smoker, N(%)** | 2 (11.1) | 1^a^ /11 (9.1) | 2 (7.1) | 1 (33.3) | 1/21 (4.8) | 0 | 0 | 0 | 3 (25) | 0 | . | 7 (28) | 0 |
| **Current Drinker, N(%)** | 4/17 (23.5) | 0 | 5 (17.9) | 1/2 (50) | 0 | 0 | 4/13 (30.8) | 0 | 0 | 4/8 (50) | . | 2/9 (22.2) | 2 (28.6) |
| **PMH** |  |  |  |  |  |  |  |  |  |  |  |  |  |
| - **HTN, N(%)** | 9 (50) | 8 (66.7) | 15 (53.6) | 2 (66.7) | 13 (59.1) | 4 (44.4) | 16 (80) | 10 (83.3) | 7 (58.3) | 5 (55.6) | 6 (46.2) | 18 (72) | 4 (57.1) |
| - **Hypercholesterolemia, N(%)** | 4 (22.2) | 6 (50) | 7 (25) | 1 (33.3) | 14 (63.6) | 5 (55.6) | 12 (60) | 7 (58.3) | 5 (41.7) | 2 (22.2) | 7 (53.9) | 13 (52) | 2 (28.6) |
| - **DM, N(%)** | 1 (5.6) | 0 | 4 (14.3) | 0 | 8 (36.4) | 1 (11.1) | 4 (20) | 2 (16.7) | 0 | 0 | 2 (15.4) | 3 (12) | 1 (14.3) |
| - **OAC, N(%)** | 1 (5.6) | 0 | 5 (17.9) | 1/2 (50) | 0 | 0 | 1/16 (6.3) | 1 (8.3) | 0 | 2 (22.2) | 1 (7.7) | 2 (8) | 1 (14.3) |
| - **Antiplatelet, N(%)** | 6 (33.3) | 3 (25) | 7 (25) | 0 | 14 (63.6) | 1 (11.1) | 2/16 (12.5) | 7 (58.3) | 6 (50) | 5 (55.6) | 5 (38.5) | 5 (20) | 2 (28.6) |
| - **Statins, N(%)** | 4 (22.2) | 3 (25) | 7 (25) | 1/2 (50) | 9 (40.9) | 2 (22.2) | 7/16 (43.8) | 6 (50) | 4 (33.3) | 1 (11.1) | 8 (61.5) | 12 (48) | 2 (28.6) |
| - **Anti HTN, N(%)** | 3 (16.7) | 7 (58.3) | 15 (53.6) | 1/2 (50) | 12 (54.6) | 3 (33.3) | 12/16 (75) | 11 (91.7) | 7 (58.3) | 4 (44.4) | 8 (61.5) | 17 (68) | 4 (57.1) |
| - **preICH, N(%)** | 0 | 3 (25) | 4 (14.3) | 0 | 1 (4.6) | 0 | 4 (20) | 0 | 2 (16.7) | 1 (11.1) | 2 (15.4) | 5 (20) | 2 (28.6) |
| - **preIS, N(%)** | 1 (5.6) | 1 (8.3) | 1 (3.6) | 0 | 1 (4.6) | 1 (11.1) | 4 (20) | 2 (16.7) | 3 (25) | 0 | 0 | 6 (24) | 1 (14.3) |
| **Future events** |  |  |  |  |  |  |  |  |  |  |  |  |  |
| **ICH, N(%)** | 2 (11.1) | 1 (8.3) | 10 (35.7) | 1 (33.3) | 9 (40.9) | 4 (44.4) | 5 (25) | 3 (25) | 1 (8.3) | 0 | 2 (15.4) | 12 (48) | 1 (14.3) |
| **Recurrent cSAH, N(%)** | 3 (16.7) | 0 | 12 (42.9) | 0 | 8 (36.4) | 0 | 3 (15) | 3 (25) | 1 (8.3) | 3 (33.3) | 0 | 6 (24) | 0 |
| **Ischemic stroke, N(%)** | 2 (11.1) | 1 (8.3) | 8 (28.6) | 0 | 1 (4.6) | 2 (22.2) | 0 | 2 (16.7) | 2 (16.7) | 0 | 0 | 1 (4) | 0 |
| **Death, N(%)** | 1 (5.6) | 1 (8.3) | 8 (28.6) | 1 (33.3) | 5 (22.7) | 1 (11.1) | 5 (25) | 2 (16.7) | 0 | 0 | 0 | 5 (20) | 2 (28.6) |

cSAH = cortical subarachnoid hemorrhage; DM = diabetes mellitus; HTN = hypertension; ICH = intracerebral hemorrhage; PMH = past medical history; preICH = previous intracerebral hemorrhage; preIS = previous ischemic stroke; OAC = oral anticoagulation; SD = standard deviation;

^a^In case of missing values in the predictors the number is displayed as a fraction and percentage is based on complete cases.

**Table e-3: characteristics of patients according to probable CAA**

| **Overall N=233** | **Possible CAA**  **N=37** | **Probable CAA**  **N=153** |
| --- | --- | --- |
| Age, mean (SD) | 75.6 (7.9) | 74.3 (9) |
| Female Sex | 13 (35.1) | 73 (47.7) |
| Current Smoker, N(%) | 3/27 (11.1) | 14/142 (9.9) |
| Current Drinker, N(%) | 5/25 (20) | 17/124 (13.7) |
| PMH |  |  |
| - HTN, N(%) | 19 (51.4) | 98 (64.1) |
| - Hypercholesterolemia, N(%) | 15 (40.5) | 70 (45.8) |
| - DM, N(%) | 6 (16.2) | 20 (13.1) |
| - OAC, N(%) | 4/33 (12.1) | 11/152 (7.2) |
| - Antiplatelet, N(%) | 8/33 (24.2) | 55/152 (36.2) |
| - Statins, N(%) | 11/33 (33.3) | 55/152 (36.2) |
| - Anti HTN, N(%) | 17/33 (51.5) | 87/152 (57.2) |
| - preICH, N(%) | 1 (2.7) | 23 (15) |
| - preIS, N(%) | 6 (16.2) | 15/152 (9.9) |
| Symptoms | 36/37 | 152/153 |
| - Negative, N(%) | 13 (36.1) | 77 (50.7) |
| - Positive, N(%) | 8 (22.2) | 50 (32.9) |
| - Both, N(%) | 8 (22.2) | 19 (12.5) |
| - Isolated Headache, N(%) | 5 (13.9) | 6 (4) |
| - None of the others, N(%) | 2 (5.6) | 0 |
| Spreading symptoms, N(%) | 6 (16.2) | 51/152 (33.6) |
| Future events |  |  |
| - ICH, N(%) | 1 (2.7) | 50 (32.7) |
| - Ischemic stroke, N(%) | 4 (10.8) | 15 (9.8) |
| - Recurrent cSAH, N(%) | 4 (10.8) | 35 (22.9) |
| Death, N(%) | 8 (21.6) | 23 (15) |
| MRI characteristics |  |  |
| - cSS | 33 (89.2) | 137/151 (90.7) |
| - CMB | 9 (24.3) | 116/150 (77.3) |
| - White matter lesions | 22/33 (66.7) | 98/143 (68.5) |

AF = atrial fibrillation; CAA = cerebral amyloid angiopathy; CMB = cerebral microbleeds; cSAH = cortical subarachnoid hemorrhage; cSS = cortical superficial siderosis; DM = diabetes mellitus; FU = follow-up; HTN = hypertension; ICH = intracerebral hemorrhage; OAC = oral anticoagulation, PMH = past medical history; preICH = previous intracerebral hemorrhage; preIS= previous ischemic stroke; SD = standard deviation;

^a^In case of missing values in the predictors the number is displayed as a fraction and percentage is based on complete case


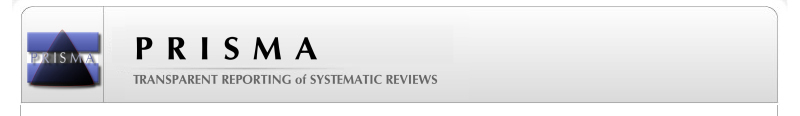
**Figure e-1. PRISMA 2009 Flow Diagram**

Records identified through database searching
(n =2243)

Records after duplicates removed
(n = 2243)

Records screened
(n =2243)

Records excluded
(n = 2222)

## Included

210 patients excluded due to not meeting inclusion criteria

7 groups did not reply or declined participation, 2 studies included same cohort

190 individuals included for main analysis

13 groups participating, 400 patients)

Full-text articles assessed for eligibility
(n = 21)

## Identification

## Eligibility

## Screening

*From:*  Moher D, Liberati A, Tetzlaff J, Altman DG, The PRISMA Group (2009). *P*referred *R*eporting *I*tems for *S*ystematic Reviews and *M*eta-*A*nalyses: The PRISMA Statement. PLoS Med 6(7): e1000097. doi:10.1371/journal.pmed1000097

**For more information, visit** [**www.prisma-statement.org**](http://www.consort-statement.org/)

**REFERENCES**

1. Wilson D, Hostettler IC, Ambler G, Banerjee G, Jager HR, Werring DJ. Convexity subarachnoid haemorrhage has a high risk of intracerebral haemorrhage in suspected cerebral amyloid angiopathy. Journal of neurology 2017.

2. Apoil M, Cogez J, Dubuc L, et al. Focal cortical subarachnoid hemorrhage revealed by recurrent paresthesias: a clinico-radiological syndrome strongly associated with cerebral amyloid angiopathy. Cerebrovascular diseases 2013;36:139-144.

3. Beitzke M, Enzinger C, Wunsch G, Asslaber M, Gattringer T, Fazekas F. Contribution of convexal subarachnoid hemorrhage to disease progression in cerebral amyloid angiopathy. Stroke; a journal of cerebral circulation 2015;46:1533-1540.

4. Beitzke M, Gattringer T, Enzinger C, Wagner G, Niederkorn K, Fazekas F. Clinical presentation, etiology, and long-term prognosis in patients with nontraumatic convexal subarachnoid hemorrhage. Stroke; a journal of cerebral circulation 2011;42:3055-3060.

5. Geraldes R, Sousa PR, Fonseca AC, Falcao F, Canhao P, Pinho e Melo T. Nontraumatic convexity subarachnoid hemorrhage: different etiologies and outcomes. Journal of stroke and cerebrovascular diseases : the official journal of National Stroke Association 2014;23:e23-30.

6. Graff-Radford J, Fugate JE, Klaas J, Flemming KD, Brown RD, Rabinstein AA. Distinguishing clinical and radiological features of non-traumatic convexal subarachnoid hemorrhage. European journal of neurology 2016;23:839-846.

7. Mas J, Bouly S, Mourand I, Renard D, de Champfleur N, Labauge P. [Focal convexal subarachnoid hemorrhage: clinical presentation, imaging patterns and etiologic findings in 23 patients]. Revue neurologique 2013;169:59-66.

8. Martinez-Lizana E, Carmona-Iragui M, Alcolea D, et al. Cerebral amyloid angiopathy-related atraumatic convexal subarachnoid hemorrhage: an ARIA before the tsunami. Journal of cerebral blood flow and metabolism : official journal of the International Society of Cerebral Blood Flow and Metabolism 2015;35:710-717.

9. Khurram A, Kleinig T, Leyden J. Clinical associations and causes of convexity subarachnoid hemorrhage. Stroke; a journal of cerebral circulation 2014;45:1151-1153.

10. Bruno VA, Lereis VP, Hawkes M, Ameriso SF. Nontraumatic subarachnoid hemorrhage of the convexity. Current neurology and neuroscience reports 2013;13:338.

11. Kumar S, Goddeau RP, Jr., Selim MH, et al. Atraumatic convexal subarachnoid hemorrhage: clinical presentation, imaging patterns, and etiologies. Neurology 2010;74:893-899.

12. Ni J, Auriel E, Jindal J, et al. The characteristics of superficial siderosis and convexity subarachnoid hemorrhage and clinical relevance in suspected cerebral amyloid angiopathy. Cerebrovascular diseases 2015;39:278-286.

13. Ly JV, Singhal S, Rowe CC, Kempster P, Bower S, Phan TG. Convexity Subarachnoid Hemorrhage with PiB Positive Pet Scans: Clinical Features and Prognosis. Journal of neuroimaging : official journal of the American Society of Neuroimaging 2015;25:420-429.

14. Refai D, Botros JA, Strom RG, Derdeyn CP, Sharma A, Zipfel GJ. Spontaneous isolated convexity subarachnoid hemorrhage: presentation, radiological findings, differential diagnosis, and clinical course. Journal of neurosurgery 2008;109:1034-1041.
